# Associations of eHealth Literacy with Social Activity among Community-Dwelling Older Adults: A Cross-Sectional Study

**DOI:** 10.3390/ejihpe14050084

**Published:** 2024-05-06

**Authors:** Mamiko Eto, Koji Yamatsu

**Affiliations:** 1 Graduate School of Medical Science, Saga University, Saga 849-8502, Japan; mamiko.eto@mark-lab.net; 2 Graduate School of Advanced Health Sciences, Saga University, Saga 849-8502, Japan; 3 Faculty of Education, Saga University, Saga 840-8502, Japan

**Keywords:** digital health literacy, eHealth Literacy Scale, sports activity, cultural activity, community activity, health behavior, frailty, elderly

## Abstract

Older adults who use digital technology are desired to adapt to digitalization and literacy. One required aspect is eHealth literacy, measured with the eHealth Literacy Scale (eHEALS). Many studies on eHealth literacy have used the eHEALS to examine the health behaviors of college students, relatively younger adults, and Internet users. However, the relevance of eHealth literacy to social activity has not yet been studied in older adults. The purposes of this study were to examine the relationship between eHealth literacy and health behaviors and social activities (community, cultural, and sports activities) and to investigate the factors associated with eHEALS scores among community-dwelling older adults. The mean eHEALS score was 12.4 points (SD 8.2), with the majority (73.3%) having the lowest score (the lowest score is 8 points). Males (17.6, SD 10.5) scored significantly higher than females (11.8, SD 7.7). The eHEALS score had a significant relationship with both cultural and community activity. Five factors significantly associated with having the lowest eHEALS score were cultural activity at least once a week, no cultural activity, no community activity, total IADL score, and intellectual activity. These results suggest that eHealth literacy is associated with community activity and cultural activity among older adults.

## 1. Introduction

The percentage of the global population aged 65 years and older is expected to increase from 10% in 2022 to 16% in 2050 [1]. The population of Japan was already 29% as of 2021 [2]. Advances in public health and medicine have helped many people to live longer and healthier lives [3]. However, not all older adults are healthy. In an aging society, healthy life expectancy is desired to extend while curbing rising healthcare expenditures without reducing the quality of healthcare services [4]. The increasing older adult population means that more people need healthcare simultaneously; this needs to be addressed using the benefits of digital technology [5]. Digital technology has great potential for addressing healthcare needs and transforming many aspects of geriatric care [6]. Older adults benefit from digital technology, including increased communication, increased activity, and improved health [7]. The COVID-19 pandemic has been a major turning point in the benefits of the use of digital technology by older adults, and the digitalization of everyday life has been accelerated. Since there is a possibility that a pandemic caused by such an infectious disease will occur in the future, it is necessary to urgently promote the acquisition and penetration of digital technology among older adults in order to be prepared. For community-dwelling older adults, digitization benefits healthcare, enhances daily life convenience, and helps to gather information during disasters [8]. Older adults need the skills and knowledge to use digital technologies [9].

Older adults are not proficient in using digital technology. According to a survey conducted by the Japan Ministry of Internal Affairs and Communications in 2020, the usage rate among older adults was low, at 86.8% for those aged 60–69, 65.5% for those aged 70–79, and 33.2% for those aged 80 and older, with over 80% of those aged 60–69 feeling uneasy about using the Internet [10]. Approximately 34% of older Internet users said they were not confident in their ability to use digital devices, and 48% said they needed someone to set up or teach them how to use them [11]. Despite the reported benefits of Internet use among older adults in improving cognitive function, social well-being [12], and loneliness [13], older adults have few opportunities to benefit from online digital technologies related to health maintenance. The disparity among older adults regarding access to and adaptation to digital technologies is known as the gray digital divide [14]. This gray digital divide constitutes a major concern shared by United Nations Economic Commission for Europe (UNECE) countries, affecting aging populations at both the individual and societal levels [15].

Older adults need eHealth literacy and digital abilities and skills to eliminate the digital divide in health. Acquiring eHealth literacy has an impact on individuals’ treatment decision-making style [16]. As eHealth literacy improves, the ability to use online health resources improves [17]. eHealth literacy is a foundational competency in using digital technologies. In increasingly digitalized healthcare, individuals must manage and utilize electronic health records and welfare service information at the individual level, allowing them to take the initiative in making choices and managing their health [4]. Therefore, minimal eHealth literacy is necessary.

eHealth literacy refers to the ability to use digital health and electronic information. This is referred to as digital health literacy. Norman and Skinner defined “the concept of eHealth literacy as the ability to seek, find, understand, and appraise health information from electronic sources and apply the knowledge gained to address or solve health problems. eHealth literacy combines facets of different literacy skills and applies them to eHealth promotion and care. At its heart are six core skills (or literacies): traditional literacy, health literacy, information literacy, scientific literacy, media literacy, and computer literacy (the Lily Model) [18]”. The eHealth Literacy Scale (eHEALS) is defined as a set of skills needed to use information technology for health effectively [19]. The eHEALS is often used as a measure of eHealth literacy [20]. The Japanese version of the eHEALS has also been developed and validated for reliability and validity [21].

Most studies on eHealth literacy have reported on health behaviors [20,22,23,24,25,26,27]. Many studies have found that eHealth literacy is associated with health behaviors, but many have focused on youth [22,23,24,25]. Despite the claimed need for digital health technologies, few studies on eHealth literacy exist for older adults. eHealth literacy is important for addressing health issues and supporting longevity through digital technologies. Therefore, this study investigated whether eHealth literacy among older adults is associated with health behaviors. Besides general health behaviors, the “Dietary Variety Score (DVS)” was added in this study. The DVS has been widely used to assess the eating behaviors of Japanese older adults [28]. The combination of food diversity, physical activity, and social interactions was highly effective in preventing disabilities in older adults [29], and ingesting various foods prevented a decrease in the independence of higher life functions of older adults living in the community [28].

eHealth literacy has also been associated with improved health status [30,31]. As decreased independence was associated with life expectancy [32], the 13 items of the Instrumental Activities of Daily Living (IADL) [33] developed by Koyano based on the Lawton assessment method [34] were employed in this study. Furthermore, we also investigated the association with frailty [35], a condition of increased vulnerability to health problems due to various age-related functional changes, and loss of reserve capacity using the revised Japanese version of the Cardiovascular Health Study (J-CHS) criteria [36]. In previous studies, the health status of older adults has mostly been determined by self-report [30,31,37]. Therefore, we adopted objective frailty assessments with confirmed validity and reliability to examine the relationship with eHealth literacy. This is a new finding, as no study has investigated an association between eHealth literacy and frailty.

In older adults, social activity effectively maintains health and improves health behaviors and statuses. Social activity prevented functional disability [38,39,40,41,42], cognitive decline [43,44], and mortality [42,45]. Although social activity has contributed to health outcomes in older adults, to our knowledge, no study has examined the association between eHealth literacy and social activities. Kanamori et al. classified social activities into eight types: neighborhood associations/senior citizen clubs/firefighting teams (local community), hobby groups (hobbies), sports groups or clubs (sports), political organizations or groups (politics), industrial or trade associations (industry), religious organizations or groups (religion), volunteer groups (volunteer), and citizen or consumer groups (citizen) [46]. Participation in local communities, hobbies, sports, or organizations may be especially effective in decreasing the risk of disability [46]. According to the Cabinet Office of Japan, social activities in older adults aged 65 and over include sports activities such as gymnastics and gateball, hobbies including haiku and shigin, and community activities including festivals [2]. These activities allow older adults to communicate with others and feel connected to society. Therefore, this study focused on three types of activities: sports, cultural activities including hobbies, and community activities. Findings from this study can provide basic information about future eHealth literacy among older adults. Moreover, the results of this study can provide basic information about eHealth literacy in the design of future health care, public policy, and programs for older adults, and can guide ways to improve eHealth literacy among older adults.

The purposes of this study were to investigate the relationship between eHealth literacy and health behaviors and social activities (community, cultural, and sports activities) among community-dwelling older adults in Japan and to identify factors associated with eHEALS scores among older adults. In particular, we hypothesized that eHealth literacy would be associated with all three types of social activities in community-dwelling older adults. Furthermore, we attempted to exploratorily examine predictors of eHealth literacy among many variables.

## 2. Research Methods

### 2.1. Study Design and Sampling

This cross-sectional study was conducted in Miyaki town, Saga Prefecture, Japan, a rural municipality with approximately 25,000 people. The participants in this study were 630 residents of Miyaki town aged 41–99 years. The recruitment method for this study was to provide information in the town newspaper, to participants in public health classes for older adults held in the town, and to general residents. eHealth literacy has never been explained or discussed in public health classes. Therefore, the target of this study was not a representative population of Miyaki town. All subjects received an explanation of the study and signed written consent. Of 630 residents, 561 aged 65 years or older were included in the analysis. Exclusion criteria were lack of consent, age 64 years or younger, missing eHEALS response items, missing social activity, health behavior, and health status items, and missing other variables. After excluding 69 participants with incomplete responses, data from 561 older adults were used for the analysis.

### 2.2. The Measurement Procedure

The measurement was conducted from October to December 2021 in 56 districts with small groups of 5 to 15 participants. The reason why the number of people to be measured was set at 5 people at a minimum and 15 people at a maximum was because the measurement location was sometimes a small community center, and consideration was given to the safety of the measurement and the waiting time of the subjects. All participants were asked to complete a questionnaire. Physical fitness and body composition were measured by trained research staff. At the end of the measurement, participants received a 10 min explanation so that they understood their own physical fitness and health status. Owing to the worldwide spread of COVID-19 in 2021, the measurement was conducted with thorough infection prevention measures in place.

### 2.3. Measurements

#### 2.3.1. eHealth Literacy Level (eHEALS)

The Japanese version of the eHEALS [21] was used. The eHEALS was developed to measure consumers’ combined knowledge, comfort, and perceived skills in finding, evaluating, and applying electronic health information to health problems [19]. The eHEALS consists of eight questions answered on a 5-point Likert scale ranging from “strongly disagree (1)” to “strongly agree (5)” for each question item. The total score of the eight items ranges from 8 [minimum] to 40 [maximum] points [21].

#### 2.3.2. Social Activity

**Figure 1:**
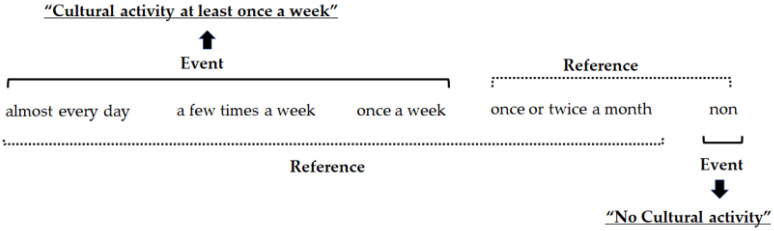
Definition when converting social activity into dummy variables.

Social activity was divided into three categories based on the study by Kanamori et al. [46]. In this study, sports activity included ground golf, gateball, walking, and community-sponsored exercise classes for health, which were mainly physical activities. Cultural activity included karaoke, dance, computer classes, Go, calligraphy, cooking classes, and senior citizen colleges. Community activity, such as neighborhood and senior citizen associations, were defined as activities that occurred in the town. Social activity was converted into dummy variables and used in the analysis. Respondents were asked to select one of the following five options for frequency of participation: “almost every day”, “a few times a week”, “once a week”, “once or twice a month”, or “non”. The responses obtained were divided into three categories: “at least once a week”, “once or twice a month”, or “non”. In cultural activity example, cultural activity can be divided into two different variables (Figure 1). The first was to calculate the odds ratio using the variable “Cultural activity at least once a week” as an event and the variable “Cultural activity less than once a week” as a reference. Second, the variable “No cultural activity” was used as an event, and “almost every day”, “at least once a week”, “once or twice a month”, and “non” are grouped together as a reference (Figure 1). 

#### 2.3.3. Health Behavior

Health behaviors were assessed using questions on balanced food intake and smoking regarding previous studies that reported associations with eHealth literacy [22,23,24,25]. In addition, going out at least once a week (yes or no) and a sitting time of at least 8 h a weekday (yes or no) were added. The Japanese government advocates nutrition, food, physical activity, and smoking as important health behaviors for improving lifestyle and reducing risk factors in the policies of the Second National Health Promotion Campaign (Healthy Japan 21) [47]. Responses were dichotomized into yes or no for smoking, physical activity, going out once a week, and sitting time of at least 8 h a weekday according to the advice most commonly offered in Japanese public health settings [48]. Physical activity adopted sports activity in social activity.

The DVS was used to assess balanced foods. The DVS was assessed by the frequency of daily food intake of 10 selected food groups: meat, seafood, eggs, milk, soy products, green and yellow vegetables, seaweed, fruits, potatoes, and fats and oils [28]. The scores range from 0 to 10, with higher scores indicating greater dietary diversity [28].

Sitting time was asked: “How much time do you usually spend at rest without physical activity, sitting or lying down every day, in total per day?” Responses were answered yes or no to “Sitting time at least 8 hours a weekday”.

#### 2.3.4. Health Status

The IADL, body mass index (BMI), and frailty were assessed. Data on self-reported disease, single fall, and multiple falls were also included in the analysis.

The IADL is a 13-item index developed by Koyano [33] based on Lawton’s evaluation method [34]. The IADL comprised five items for “Instrumental self-maintenance”, four items for “Intellectual activity”, and four items for “Social role”. The 13 questions were self-administered and required a “yes (1 point)” or “no (0 point)” response; the higher the score, the more independent the IADL. The IADL refers to complex activities in daily life, such as shopping, coordination, laundry, telephone, medication management, property management, use of public transportation, etc. [47].

BMI was calculated as the weight divided by the height squared (kg/m^2^) from actual height and weight measurements. BMI was used as a proxy for long-term health behavior. We categorized participants as “obesity (≥25 kg/m^2^)” or “non-obesity” based on BMI. We also used separate classifications of “thin (<18.5 kg/m^2^)” or “non-thin” [49].

The J-CHS criteria were used as the diagnostic criteria for frailty. The J-CHS is based on the Fried phenotype model [50], modified for older Japanese individuals. The J-CHS includes five elements: (1) unintentional weight loss, (2) weakness (grip strength), (3) exhaustion, (4) slowness (normal gait speed), and (5) low physical activity [36]. (1) Unintentional weight loss was defined as a “yes” response to the question “Did you unintentionally lose 2 to 3 kg or more in 6 months?”. (2) Weakness was assessed using grip strength. Grip strength was measured by having one person grip the measuring device twice on each side with maximum effort. A maximum of four grip strength values was used for the analysis. The cutoff values were 28 kg for males and 18 kg or less for females. (3) Exhaustion was defined as a “yes” response to the question “I feel tired for no reason for the past two weeks?”. (4) Slowness was assessed using gait speed. The participants were asked to walk a distance of 11 m once at their usual walking speed. The measuring staff used a stopwatch to measure the time required to move 5 m on the way from the 3 m to the 8 m point. Normal gait speed was calculated as “5 m divided by the number of seconds required to move”. (5) Low physical activity was defined as “Do you do light exercise or physical training?” and “Do you engage in regular exercise/sports?”, and the two questions were asked. Both questions were applied to those who answered “no”. In the J-CHS criteria, we categorized cases that apply answered “no” to all five items as “robust”, cases that apply to one or two items as “pre-frailty”, and cases that apply to three or more items as “frailty” [36].

Regarding pre-existing medical conditions, we asked if there were any diseases being treated and the types of diseases. When asked if they had fallen within the past year, those who answered no were treated as “no”, those who had fallen once were treated as “single fall”, and those who had fallen more than once were treated as “multiple falls”.

## 3. Statistical Analysis

Comparisons of gender differences in participant characteristics were analyzed using Student’s *t*-test or the chi-square test.

The association between the eHEALS and social activities was verified using logistic regression analysis. The independent variable was the eHEALS, which was used here as a continuous variable. The dependent variable was social activity, which is defined as a dummy variable. The associations between the eHEALS and health behaviors (dummy variable) or health status (dummy variable) were analyzed in the same way. Odds ratio (OR) and 95% confidence interval (95%CI) were calculated with logistic regression analysis.

In verifying the predictors of the eHEALS, 73.3% of all subjects had the lowest eHEALS score of 8 points, so a logistic regression analysis was performed using the lowest eHEALS score (dummy variable) as the dependent variable. At that time, those who did not have the lowest eHEALS score were used as reference. Because this analysis was an exploratory study, we examined a number of factors that could predict who would score the lowest on the eHEALS.

Logistic regression analysis first examined the association between the eHEALS and other factors (such as social activity) but ultimately statistically adjusted for age and gender (with males as the reference).

The significance level was set at less than 5%.

## 4. Results

### 4.1. Baseline Characteristics

The overall mean age was 76.9 years (SD 5.9), with males having a mean age of 75.3 years (SD 6.2) and females having a mean age of 77.1 years (SD 5.8), and females had a significantly higher mean age (*p* < 0.05) (Table 1). Participants were 561 older adults: 60 (10.7%) males and 501 (89.3%) females. The age distribution of the participants was 13 in the 65–69 age group, 335 in the 70–79 age group, 198 in the 80–89 age group, and 15 aged 90 or above.

In social activity, males were more likely than females to be involved in community activity at least once a week. There were no gender differences in sports activity and cultural activity. Sports activity was performed at least once a week by 356 respondents (63.5%). Cultural activity was answered “non” by 408 (72.7%) respondents.

The DVS had a mean score of 6.2 (SD 2.4). The DVS was 4.9 (SD 2.5) among males and 6.3 (SD 2.3) among females. Females’ DVS was significantly higher than males (*p* < 0.001).

Regarding smoking habits, 548 participants (97.7%) were nonsmokers. Regarding the presence of diseases, 493 participants (87.9%) reported having chronic conditions. The types of diseases included 16 cases of cancer, 58 cases of heart disease, 5 cases of stroke, 238 cases of hypertension, 62 cases of diabetes, 22 cases of obesity, 169 cases of hyperlipidemia, 154 cases of osteoporosis, 73 cases of joint pain or neuropathy, 16 cases of injury or fracture, 40 cases of respiratory disease, 14 cases of gastrointestinal disease, and 4 cases of liver disease. However, no significant correlation was observed between the number of diseases and the eHEALS score.

Other characteristics of the subjects are also shown in the table (Table 1).

### 4.2. eHealth Literacy Level

The mean eHEALS score was 12.4 points (SD, 8.2) (Table 2). Males, at 17.6 points (SD 10.5), scored higher than females, at 11.8 points (SD 7.7) (*p* < 0.001). The mean scores for each eHEALS item ranged from 1.5 (0.9) to 1.6 points (1.2) (males: 2.1–2.3, females: 1.4–1.5). There were no differences in most questionnaire items. The eHEALS scores were significantly higher for males across all eight items (*p* < 0.001). From the reliability statistics, Cronbach’s alpha for eight items in the eHEALS was 0.985.

Overall, the number of participants with the lowest eHEALS score (8 points) was the majority (73.3%). Females (76.4%) were significantly more likely to have a lower eHEALS score than males (46.7%, *p* < 0.001). The highest eHEALS score (40 points) was obtained in four female participants.

### 4.3. Association of eHEALS Score with Social Activity

As a result of logistic regression analysis, significant associations were found between eHEALS score and two types of social activities (Table 3). The eHEALS score was positively associated with cultural activity at least once a week (OR: 1.037, 95%CI: 1.007–1.068) and negatively associated with no cultural activity (OR: 0.955, 95%CI: 0.933–0.977). Also, the eHEALS score was positively associated with community activity at least once a week (OR: 1.031, 95%CI: 1.003–1.059) and negatively associated with no community activity (OR: 0.971, 95%CI: 0.949–0.994). Regarding sports activity, no association with eHEALS score was observed.

### 4.4. Association of eHEALS Score with Health Behavior

eHEALS score was not associated with the DVS (Table 4). There were no associations between eHealth literacy and health behaviors.

### 4.5. eHEALS Score and Health Status

The eHEALS score had significant relationships between the IADL total score and intellectual activity in IADL (Table 4). The eHEALS score was positively related to the IADL total score (OR: 1.032, 95%CI: 1.006–1.058) and intellectual activity (OR: 1.040, 95%CI: 1.006–1.074). The eHEALS score was not significantly related to other health status variables.

### 4.6. Associated Factors with the Lowest eHEALS Score

The factors associated with having the lowest eHEALS score are shown (Table 5). Five items were found to have a significant relationship with the lowest eHEALS score: cultural activity at least once a week, no cultural activity, no community activity, the IADL total score, and intellectual activity.

Engaging in cultural activity once a week had a favorable influence on having the lowest eHEALS score, which was reduced by almost half (OR: 0.540, 95%CI: 0.307–0.948). “No cultural activity” had a negative influence, and was approximately 1.8 times higher than the lowest eHEALS score (OR, 1.845; 95%CI: 1.192–2.856). “No community activity” had a negative association, and had approximately a twofold increase in the lowest eHEALS score (OR: 1.946, 95%CI: 1.276–2.967). After statistically adjusting for age and gender, “no community activity” was still significant and community activity more than once a week was, oppositely, not significant.

Older adults with higher IADL total scores were less likely to have the lowest eHEALS score (OR, 0.819; 95%CI: 0.679–0.989). Intellectual activity also had a favorable influence on having the lowest eHEALS score (OR, 0.568; 95%CI: 0.377–0.855). Significant associations were found for some health statuses (ex, social role and robust), but all variables were no longer significant after adjusting for age and gender.

Finally, in order to clarify which was the most powerful predictor, we performed multivariate logistic regression analysis using the variables in Table 5 that were significant even after adjusting for gender and age. “Cultural activity at least once a week” and “No cultural activity” were entered separately. As a result of inserting “cultural activity at least once a week”, “no community activity”, and “IADL total score” as independent variables (gender and age were also included as adjustment factors), only “no cultural activity” was significant. The OR for “no community activity” was 1.838 (95%CI: 1.201–2.814). Next, we entered “no cultural activity”, “no community activity”, and “IADL total score” as independent variables (gender and age were also included as adjustment factors). As a result, “no community activity” and “no cultural activity” were significant. The OR for “no community activities” was 1.780 (95%CI: 1.159–2.734) and the OR for “no cultural activities” was 1.595 (95%CI: 1.020–2.495).

## 5. Discussion

### 5.1. eHealth Literacy Level

The mean eHEALS score in this study was low at 12.4 points (SD 8.2). This score was relatively lower than 21.0 points for older adults (mean age 73.6) living in Shizuoka, Japan [51]. Another previous study reported the eHEALS scores of older adults overseas as follows: the US older adults mean of 24.5 points [52], Sweden’s older adults mean of 27.5 points [37], and the Chinese older adults mean of 17.2 points [26]. The eHEALS scores in this study were relatively lower than those reported in all previous studies. It was assumed that participants in this study were highly health conscious because they wanted to participate in this study and monitor their physical fitness and health status. But, they had lower eHEALS scores. The reason for this lower eHealth literacy level was unknown, but the possibility that the “digital divide” due to age had an influence cannot be denied. The Japanese government is concerned about the negative impact of the digital divide on older adults [53], so it will be necessary to continue research on this issue.

### 5.2. Association between eHEALS and Social Activity

eHealth literacy was associated with two types of social activities. Firstly, eHealth literacy was associated with cultural activities (“cultural activities at least once a week” and “no cultural activity”). Cultural activity is often related to hobbies and may involve using the Internet to obtain information on materials and methods for tackling new tasks. Using the Internet and other means of signing up for new classes may also be related to eHealth literacy. Although it is unclear why there was a significant relationship between eHealth literacy and cultural activity, we hope that future longitudinal studies will explain the mechanisms underlying this relationship.

Secondly, eHealth literacy has also been associated with community activity (“community activity at least once a week” and “no community activity”). Community activity in this study was organizational activities unique to Japan, such as residents’ associations and senior citizens’ clubs. A residents’ association is an organization formed based on the local connections of people between people who live in a specific area within a municipality [54]. A senior citizens’ club is an organization that conducts projects to maintain and promote the physical and mental health of older adults based in the community under the “Welfare Law for Older Adults”, enacted in 1963 [55]. One possible reason for this may be that community activities facilitate access to various types of information because information from Japanese administrative agencies such as local government welfare departments is actively provided when community activities collaborate with public agencies. Another possible reason may be the impact of social activity on health and health behaviors. Social activity has been reported to prevent frailty and the need for nursing care; in particular, participation in local communities and hobbies was effective [40,46]. Although some aspects remain unclear about the mechanisms that prevent functional impairment in social activity, engaging in social activity may contribute to increasing the activeness and social capital of older adults because social activity encourages older adults to go out and be more active in their daily lives and may increase social support by frequently access to health information and communication with neighbors [46]. Therefore, it is possible that eHealth literacy is related to social activity, such as cultural and community activities, among older adults in the community.

This study showed no significant association between eHealth literacy and sports activity. Sports activity is also an effective health behavior that prevents functional impairment by increasing physical activity [46,56]. A previous study of young people found an association between regular exercise and eHealth literacy [22,23,24,25]. eHealth literacy was hypothesized to be related to sports activity, but no association was found. We found that 63.5% of the participants engaged in sports activities at least once a week, which was the most common activity among social activities. In addition, most participants were a highly health-conscious group who attended health classes on their own and engaged in other sports activities. Therefore, it is possible that no association with sports activity was observed.

### 5.3. Association between eHEALS and Health Behavior or Health Status

This study found no association between eHealth literacy and health behaviors. We examined health behaviors such as smoking, eating, going out at least once a week, and a sitting time of at least 8 h a weekday. The eHEALS scores were not associated with smoking status. These results were consistent with general surveys of Japanese adults [27] and university students [22]. We cannot determine the reason for this, but it may be because there were many nonsmokers, and the mean age was high.

The results of this study showed associations between eHealth literacy and IADL total score and intellectual activity in older adults. But, there was no association with frailty variables in this study. Previous studies have reported that health literacy had association with frailty [57]. Although health literacy is also included in the concept of eHealth literacy, no study has observed the relationship between eHealth literacy and frailty. This study is the first to examine these relationships and can be said to be original.

### 5.4. Factors Associated with the Lowest eHEALS Score

Five factors significantly associated with having the lowest eHEALS score were cultural activity at least once a week, no cultural activity, no community activity, total IADL score, and intellectual activity.

Engaging in cultural activity at least once a week had a favorable influence (decrease in the risk) and “no cultural activity” had a negative influence (increase in the risk) on having the lowest eHEALS score. These results suggest that cultural activity may be a predictive factor of the lowest level of eHealth literacy.

“No community activity” was found to be associated with having the lowest eHEALS score. Community activity has the advantage of providing social support, such as access to health-related information [46], and eHealth literacy may be affected by the lack of access to such opportunities. “Cultural activity at least once a week” and “no cultural activity” were associated with eHealth literacy, while only “no cultural activity” was associated with community activity after adjusting age and gender. Regarding the difference between cultural activity and community activity, we can also consider that one of the influences is that community activities have negative aspects, such as obligations and burdens to the community [58].

Higher IADL scores had a lower likelihood of resulting in the lowest eHEALS score. Some degree of independence and cognitive function may be required to use the Internet to operate digital technologies. Therefore, higher levels of IADL may have an association with eHealth literacy.

### 5.5. Comparison with Previous Studies and Key Findings

A new finding of this study was the association between eHealth literacy and social activities (cultural activities and community activities). These results show that (1) participating in community activities at least once a week is the best choice, and (2) not participating in cultural or community activities at all may reduce the risk of having the lowest level of eHealth literacy. To our knowledge, no previous studies have investigated the association between eHealth literacy and social activities in older adults. This study may add a new perspective to eHealth literacy research. In future digitalized society, social activity may be useful in programs that reduce elderly adults’ concerns about digital technology and technology. Finding the causal relationship between eHealth literacy and social activities is desired. On the other hand, the relationship between eHealth literacy and health behaviors observed in previous studies was not observed at all [22,23,24,25,59], but the reason for the discrepancy between the results of this study and previous studies is unclear. The mean eHEALS score of participants in this study was low, at 12.4 out of 40 (SD 8.2), which may be a contributing factor. Future research will need a longitudinal study to determine whether the initiation of cultural or community activities increases eHealth literacy among participants with low eHealth literacy. The current study also investigated the association between eHealth literacy and health status (IADL, frailty, DVS, etc.) in older adults. Although no association was found in the results of this study, these are items that have not been investigated in previous eHealth literacy studies. Future research will provide basic research for developing public policies, medical practices, and programs for older adults, including improving eHealth literacy among older adults, promoting digital health, and bridging the digital divide.

### 5.6. Limitations

This study had several limitations. First, the participants were from only one town in a rural area. The results may not be applicable to older adults in other regions. Second, this study did not measure individual characteristics related to socio-economic status or the Internet environment. In particular, the influence of educational background and academic background is important, so future research should evaluate it as much as possible. Third, the causal relationship between eHealth literacy and the variables in this study is unclear because this study was cross-sectional. Finally, this study was limited to those who attended health classes, which may have been influenced by the fact that they were more health conscious than the general older adult population.

## 6. Conclusions

This study investigated the association between eHealth literacy and social activities among community-dwelling older adults. As a result, eHealth literacy was significantly associated with both cultural activity and community activity. The eHealth literacy level of older adults in this study was relatively very low. Engaging in cultural activity had positive influences on the risk of having the lowest levels of eHealth literacy. Also, interestingly, avoiding “no cultural activity” and “no community activity” had positive influences. This study is one of the first to show the relationship between eHealth literacy and social activity among older adults. The results of this study add a new perspective to previous eHealth literacy research.

## Figures and Tables

**Table 1:** Participant characteristics.

	Characteristic	Total (n = 561)		Male (n = 60)		Female (n = 501)		
		Mean	(SD)	Mean	(SD)	Mean	(SD)	*p*
Age (years)		76.9	(5.9)	75.3	(6.2)	77.1	(5.8)	0.024
Dietary Variety Score (points)		6.2	(2.4)	4.9	(2.5)	6.3	(2.3)	<0.001
IADL Total score (points)		12.2	(1.5)	12.2	(1.4)	12.2	(1.5)	0.876
Instrumental Self-maintenance (points)		4.9	(0.6)	4.9	(0.6)	4.9	(0.6)	0.870
Intellectual Activity (points)		3.7	(0.7)	3.7	(0.7)	3.7	(0.7)	0.607
Social Role (points)		3.6	(0.8)	3.6	(0.7)	3.6	(0.8)	0.790
Body Mass Index (kg/m^2^)		23.2	(3.3)	24.3	(2.9)	23.1	(3.3)	0.010
Grip strength (kg)		26.3	(6.7)	39.6	(6.9)	24.8	(4.7)	<0.001
Gait Speed (m/s)		1.30	(0.27)	1.34	(0.29)	1.29	(0.27)	0.251
	**Characteristic**	**n**	**(%)**	**n**	**(%)**	**n**	**(%)**	***p***
Gender	Male	60	(10.7)					
Female		501	(89.3)					
**Social Activity**								
Cultural Activity	at least once a week	73	(13.0)	7	(11.7)	66	(13.2)	0.327
	once or twice a month	80	(14.3)	5	(8.3)	75	(15.0)	
	non (no activity)	408	(72.7)	48	(80.0)	360	(71.9)	
Community Activity	at least once a week	91	(16.2)	22	(36.7)	69	(13.8)	<0.001
	once or twice a month	206	(36.7)	27	(45.0)	179	(35.7)	
	non (no activity)	264	(47.1)	11	(18.3)	253	(50.5)	
Sports Activity	at least once a week	356	(63.5)	39	(65.0)	317	(63.3)	0.320
	once or twice a month	185	(33.0)	17	(28.3)	168	(33.5)	
	non (no activity)	20	(3.6)	4	(6.7)	16	(3.2)	
**Health Behavior**								
Going out at least once a week	Yes	553	(98.6)	60	(100.0)	493	(98.4)	0.324
	No	8	(1.4)	0	(0.0)	8	(1.6)	
Sitting time at least 8 hours a weekday	Yes	20	(3.6)	2	(3.3)	18	(3.6)	0.918
	No	541	(96.4)	58	(96.7)	483	(96.4)	
Smoking	Yes	13	(2.3)	3	(5.0)	10	(2.0)	0.144
	No	548	(97.7)	57	(95.0)	491	(98.0)	
**Health Status**								
Obesity	Yes	155	(27.6)	23	(38.3)	132	(26.3)	0.050
	No	406	(72.4)	37	(61.7)	367	(73.3)	
Thin	Yes	40	(7.1)	2	(3.3)	38	(7.6)	0.227
	No	521	(92.9)	58	(96.7)	463	(92.4)	
Robust	Yes	364	(64.9)	39	(65.0)	325	(64.9)	0.984
	No	197	(35.1)	21	(35.0)	176	(35.1)	
Pre-frailty	Yes	185	(33.0)	21	(35.0)	164	(32.7)	0.724
	No	376	(67.0)	39	(65.0)	337	(67.3)	
Pre-frailty or Frailty	Yes	197	(35.1)	21	(35.0)	176	(35.1)	0.984
	No	364	(64.9)	39	(65.0)	325	(64.9)	
Weakness	Yes	32	(5.7)	1	(13.3)	31	(11.4)	0.154
	No	529	(94.3)	59	(86.7)	470	(88.6)	
Slowness	Yes	67	(11.9)	6	(10.0)	61	(12.2)	0.623
	No	494	(88.1)	54	(90.0)	440	(87.8)	
Unintentional Weight loss	Yes	65	(11.6)	8	(13.3)	57	(11.4)	0.655
	No	496	(88.4)	52	(86.7)	444	(88.6)	
Exhaustion	Yes	82	(14.6)	8	(13.3)	74	(14.8)	0.766
	No	479	(85.4)	52	(86.7)	427	(85.2)	
Low physical activity	Yes	37	(6.6)	5	(8.3)	32	(6.4)	0.566
	No	524	(93.4)	55	(91.7)	469	(93.6)	
Desease	Yes	493	(87.9)	51	(85.0)	442	(88.2)	0.470
	No	68	(12.1)	9	(15.0)	59	(11.8)	
Single fall	Yes	146	(26.0)	11	(18.3)	135	(26.9)	0.151
	No	415	(74.0)	49	(81.7)	369	(73.7)	
Multiple falls	Yes	34	(6.1)	2	(3.3)	32	(6.4)	0.349
	No	527	(93.9)	58	(96.7)	469	(93.6)	

**Table 2:** The eHEALS scores in this study.

	Characteristic	Total (n = 561)		Male (n = 60)		Female (n = 501)		
		Mean	(SD)	Mean	(SD)	Mean	(SD)	*p*
eHEALS (points)		12.4	(8.2)	17.6	(10.5)	11.8	(7.7)	<0.001
Q1. I know what health resources are available on the internet.		1.6	(1.2)	2.3	(1.4)	1.5	(1.1)	<0.001
Q2. I know where to find helpful health resources on the internet.		1.6	(1.1)	2.3	(1.4)	1.5	(1.1)	<0.001
Q3. I know how to find helpful health resources on the internet.		1.6	(1.1)	2.2	(1.5)	1.5	(1.1)	<0.001
Q4. I know how to use the internet to answer my questions about health.		1.6	(1.1)	2.3	(1.4)	1.5	(1.1)	<0.001
Q5. I know how to use the health information I find on the internet to help me.		1.6	(1.1)	2.2	(1.4)	1.5	(1.0)	<0.001
Q6. I have the skills I need to evaluate the health resources I find on the internet.		1.5	(1.0)	2.2	(1.4)	1.4	(0.9)	<0.001
Q7. I can tell high-quality health resources from low-quality health resources on the internet.		1.5	(0.9)	2.1	(1.3)	1.4	(0.9)	<0.001
Q8. I feel confident in using information from the Internet to make health decisions.		1.5	(1.0)	2.1	(1.3)	1.4	(0.9)	<0.001
	**Characteristic**	**n**	**(%)**	**n**	**(%)**	**n**	**(%)**	***p***
eHEALS Score	8 (Lowest point)	411	(73.3)	28	(46.7)	383	(76.4)	<0.001
	9–40 (point)	150	(26.7)	32	(53.3)	118	(23.6)	

**Table 3:** Relationship between eHEALS (continuous variable) and Social Activity.

Social Activity	Unadjusted OR (95%CI)	Adjusted ^$^ OR (95%CI)
Cultural Activityat least once a week (No)	1.029 (1.002–1.057) *	1.037 (1.007–1.068) *
No Cultural Activity (At lesat once or twice a month)	0.962 (0.942–0.983) *	0.955 (0.933–0.977) *
Community Activity at least once a week (No)	1.040 (1.015–1.065) *	1.031 (1.003–1.059) *
No Community Activity (At lesat once or twice a month)	0.959 (0.938–0.980) *	0.971 (0.949–0.994) *
Sports Activity at least once a week (No)	1.020 (0.998–1.042)	1.019 (0.995–1.043)
No Sports Activity (At lesat once or twice a month)	1.010 (0.960–1.063)	1.009 (0.954–1.068)

OR, odds ratio; CI, confidence interval; * *p* < 0.05; ^$^ Adjusted for age and gender; The notation in parentheses indicates a reference.

**Table 4:** Relationships between eHEALS (continuous variable) and Health Behavior or status.

	Unadjusted OR (95%CI)	Adjusted ^$^ OR (95%CI)
**Health Behavior**		
Dietary Variety Score ^#^	0.993 (0.973–1.014)	1.005 (0.983–1.028)
Going out at least once a week (No)	1.101 (0.919–1.318)	1.032 (0.862–1.235)
Sitting time at least 8 h a weekday (No)	0.970 (0.908–1.037)	0.960 (0.894–1.031)
Smoking (Yes)	1.016 (0.956–1.080)	1.004 (0.939–1.075)
**Health Status**		
IADL Total score ^#^	1.045 (1.020–1.069) *	1.032 (1.006–1.058) *
Instrumental Self-maintenance ^#^	1.072 (1.013–1.134) *	1.044 (0.984–1.107)
Intellectual Activity ^#^	1.049 (1.017–1.083) *	1.040 (1.006–1.074) *
Social Role ^#^	1.031 (1.004–1.059) *	1.021 (0.992–1.051)
Body Mass Index ^#^	0.999 (0.979–1.019)	0.988 (0.966–1.010)
Obesity (No)	1.007 (0.985–1.029)	0.998 (0.974–1.022)
Thin (No)	0.995 (0.956–1.036)	1.008 (0.966–1.052)
Robust (No)	1.032 (1.009–1.057) *	1.016 (0.991–1.042)
Pre-frailty (No)	0.973 (0.951–0.996) *	0.985 (0.960–1.010)
Pre-frailty or Frailty (No)	0.969 (0.946–0.991) *	0.984 (0.960–1.009)
Weakness (No)	0.924 (0.856–0.998) *	0.967 (0.894–1.046)
Slowness (No)	0.944 (0.903–0.988) *	0.985 (0.939–1.033)
Unintentional Weight loss (No)	0.977 (0.943–1.013)	0.983 (0.946–1.022)
Exhaustion (No)	0.962 (0.928–0.997) *	0.975 (0.939–1.012)
Low physical activity (No)	1.016 (0.979–1.054)	1.012 (0.972–1.054)
Desease (No)	0.998 (0.968–1.029)	0.998 (0.965–1.031)
Single fall (No)	0.978 (0.954–1.003)	0.985 (0.960–1.012)
Multiple falls (No)	0.984 (0.940–1.031)	0.999 (0.951–1.050)

OR, odds ratio; CI, confidence interval; * *p* < 0.05; ^$^ Adjusted for age and gender; ^#^ Used below average value as reference. The notation in parentheses indicates a reference.

**Table 5:** Predictors of Being Lowest eHEALS Score.

	Unadjusted OR (95%CI)	Adjusted ^$^ OR (95%CI)
Age ^#^	1.142 (1.099–1.186) *	
Gender (Male)	3.709 (2.145–6.414) *	
**Social Activity**		
Cultural Activity at least once a week (No)	0.616 (0.366–1.036) *	0.540 (0.307–0.948) *
No Cultural Activity (At lesat once or twice a month)	1.699 (1.135–2.544) *	1.845 (1.192–2.856) *
Community Activity at least once a week (No)	0.549 (0.342–0.882) *	0.650 (0.386–1.095)
No Community Activity (At lesat once or twice a month)	2.354 (1.587–3.490) *	1.946 (1.276–2.967) *
Sports Activity at least once a week (No)	0.761 (0.512–1.132)	0.763 (0.499–1.166)
No Sports Activity (At lesat once or twice a month)	0.846 (0.319–2.244)	0.849 (0.293–2.462)
**Health Behavior**		
Dietary Variety Score ^#^	1.018 (0.942–1.100)	0.957 (0.877–1.044)
Going out at least once a week (No)	0.912 (0.182–4.569)	2.812 (0.527–15.00)
Sitting time at least 8 h a weekday (No)	1.478 (0.486–4.495)	1.848 (0.535–6.380)
Smoking (No)	0.817(0.248–2.694)	1.015(0.276–3.725)
**Health Status**		
IADL Total Score ^#^	0.750 (0.626–0.899) *	0.819 (0.679–0.989) *
Instrumental Self-maintenance ^#^	0.447 (0.227–0.880) *	0.585 (0.303–1.129)
Intellectual Activity ^#^	0.518 (0.347–0.772) *	0.568 (0.377–0.855) *
Social Role ^#^	0.836 (0.644–1.086)	0.953 (0.714–1.273)
Body Mass Index ^#^	0.987 (0.933–1.045)	1.032 (0.970–1.097)
Obesity (No)	0.975 (0.643–1.480)	1.193 (0.757–1.879)
Thin (No)	1.102 (0.525–2.314)	0.842 (0.388–1.829)
Robust (No)	0.588 (0.389–0.889) *	0.740 (0.473–1.156)
Pre-frailty (No)	1.568 (1.033–2.379) *	1.327 (0.846–2.083)
Frailty or Pre-frailty (No)	1.700 (1.125–2.569) *	1.352 (0.865–2.113)
Weakness (No)	3.720 (1.116–12.40) *	1.918 (0.549–6.701)
Slowness (No)	2.574 (1.242–5.335) *	1.322 (0.603–2.895)
Unintentional Weight loss (No)	1.703 (0.883–3.283)	1.758 (0.842–3.671)
Exhaustion (No)	1.602 (0.896–2.866)	1.233 (0.665–2.288)
Low physical activity (No)	0.654 (0.324–1.319)	0.713 (0.330–1.544)
Disease (No)	1.163 (0.665–2.034)	1.160 (0.634–2.123)
Single fall (No)	1.573 (0.999–2.477) *	1.397 (0.863–2.262)
Multiple falls (No)	1.755 (0.712–4.325)	1.514 (0.572–4.008)

OR, odds ratio; CI, confidence interval; * *p* < 0.05; ^$^ Adjusted for age and gender; ^#^ Used as a continuous variable. The notation in parentheses indicates a reference.

## Data Availability

The data presented in this study are available on request from the corresponding author.
